# Effects of implant length and 3D bone-to-implant contact on initial stabilities of dental implant: a microcomputed tomography study

**DOI:** 10.1186/s12903-017-0422-1

**Published:** 2017-11-21

**Authors:** Jui-Ting Hsu, Aaron Yu-Jen Wu, Lih-Jyh Fuh, Heng-Li Huang

**Affiliations:** 1 School of Dentistry, China Medical University, 91 Hsueh-Shih Road, Taichung, 40402 Taiwan; 2 Department of Bioinformatics and Medical Engineering, Asia University, 500 Lioufeng Rd, Wufeng Taichung, 41354 Taiwan; 3 Department of Dentistry, Chang Gung Memorial Hospital & College of Medicine, Chang Gung University, 123 Ta-Pei Road, Niao-Sung Kaohsiung, 83305 Taiwan

**Keywords:** Microcomputed tomography, Implant length, 3D Bic%, Insertion torque, Periotest, Implant stability quotient

## Abstract

**Background:**

The influences of potential bone-to-implant contact (BIC) area (pBICA), BIC area (BICA), and three dimensional (3D) BIC percentage (3D BIC%; defined as BICA divided by pBICA) in relation to the implant length on initial implant stability were studied. Correlations between these parameters were also evaluated.

**Methods:**

Implants with lengths of 8.5, 10, 11.5, and 13 mm were placed in artificial bone specimens to measure three indexes of the initial implant stability: insertion torque value (ITV), Periotest value (PTV), and implant stability quotient (ISQ). The implants and bone specimens were also scanned by microcomputed tomography, and the obtained images were imported into Mimics software to reconstruct the 3D models and calculate the parameters of 3D bone-to-implant contact including pBICA, BICA, and 3D BIC%. The Kruskal-Wallis test, Wilcoxon rank-sum test with Bonferroni adjustment, and Spearman correlations were applied for statistical and correlation analyses.

**Results:**

The implant length affected ITV more than PTV and ISQ, and significantly affected pBICA, BICA, and 3D BIC%. A longer implant increased pBICA and BICA but decreased 3D BIC%. The Spearman coefficients were high (>0.78) for the correlations between the three 3D BIC parameters and the three indexes of the initial implant stability.

**Conclusions:**

pBICA, BICA, and 3D BIC% are useful when deciding on treatment plans related to various implant lengths, since these 3D BIC parameters are predictive of the initial implant stability.

## Background

The initial stabilization of a dental implant is considered to be a crucial factor influencing implantation success [1] and the development of osseointegration between the implant and bone [2]. Inadequate initial implant stability may allow micromovement between the implant and bone that results in the formation of fibrous tissue ingrowth into the interface instead of osseointegration [3]. Especially for patients with jaw bone of poor quality, the dimensions of the implant (length or diameter) are important factors in anchoring the implant strongly in bone [4–6] and hence preventing its initial mobility.

A shorter implant will generally have less contact with the surrounding bone, which could result in lower initial implant stability [6–8]. However, the results from studies of the relationship between initial implant stability and implant length have been inconsistent, with Hong et al. [6] finding that the initial implant stability was influenced by the implant length, Degidi et al. [9] indicating that only a weak correlation existed between initial implant stability and implant length, and Ostman et al. [10] reporting that a longer implant is associated with a lower initial implant stability.

While increasing the implant length would be expected to increase the surface area of an implant, the additional (deeper) portion of the implant length is only in contact with trabecular bone, which has a highly porous structure. This means that the actual increase in contact area between the implant and bone might be less than expected for a longer implant. Therefore, if clinicians want to increase the initial implant stability by embedding the longer implant in bone for patients, the measurement of actual contact area between the implant and bone seems to be necessary, especially for the three-dimensional (3D) bone-to-implant contact (BIC) [8, 11].

Cone-bean computer tomography (CBCT) is widely accepted as a useful tool in the diagnosis of diseases, injuries, and defects in oral and maxillofacial regions [12]. The advantages of CBCT over the conventional computed tomography include it involving smaller and cheaper hardware, producing higher image accuracy focused on a localized area, rapid scan time, dose reduction, and smaller image artifacts when using artifact suppression algorithms supplied by the device manufacturers [13]. CBCT has been increasingly applied in treatment planning for computer assisted maxillofacial surgery and dental implantation [14], such as for determining the available dimension (e.g., height and width) and quality of alveolar bone [15, 16], identifying anatomical structures (e.g., inferior alveolar nerve, mental foramen, and maxillary sinus) [17] and assessing bone grafting [18, 19]. Although CBCT is useful for dentists and oral surgeons, limitations in its resolution and in the functionality of software supplied with CBCT devices mean that 3D BIC still cannot be obtained for use in diagnoses nowadays. However, recent developments in micro-level CBCT images and professional medical imaging technology has allowed areas of 3D BIC and surfaces of implant site to be evaluated as parameters correlated with the initial stability of an implant [8, 11, 20, 21].

The objective of this study was to determine the impact of implant length on the initial stability of implants. In addition, high-resolution microcomputed tomography (micro-CT) images were used to calculate the following three types of 3D BIC parameter and evaluate how these parameters influence the initial implant stability: the potential bone-to-implant contact area (pBICA) [22], which is the total exterior surface area of the dental implant inside the artificial bone specimen; the actual area of contact between the bone and implant (BICA); and the 3D BIC percentage (3D BIC%), which was calculated as BICA divided by pBICA [8, 11, 20].

## Methods

### Preparation of artificial bone specimens and dental implants

**Fig. 1:**
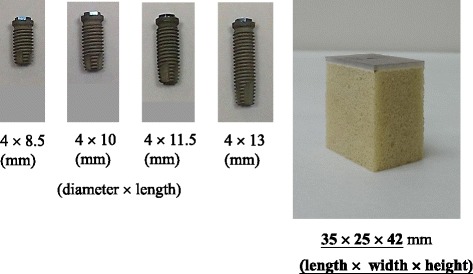
(**a**) Dental implants with four lengths (8.5, 10, 11.5, and 13 mm) and (**b**) an artificial bone specimen comprising a cortical layer and the cellular structure of trabecular bone

Rigid cellular polyurethane blocks (Sawbones, Vashon, WA, USA) representing trabecular bone with an elastic modulus of 137 MPa (model 1522–12) were attached to a 2-mm-thick synthetic cortical shells (model 3401–01) with an elastic modulus of 16.7 GPa (Fig. 1). The structure of cellular polyurethane artificial bone is porous, which simulated the human trabecular bone structure. Commercial dental implants with the same diameter (4 mm; ICE, 3i Implant Innovation, Palm Beach, FL, USA) and four different lengths were used in this study: 8.5, 10, 11.5, and 13 mm (models XFOS 485, XFOS 410, XFOS 411, and XFOS 413, respectively) (Fig. 1). Twenty specimens divided into four types of experimental group were prepared for measurements of initial implant stability indexes and 3D BIC parameters.

### Measurement of implant initial stability

**Fig. 2:**
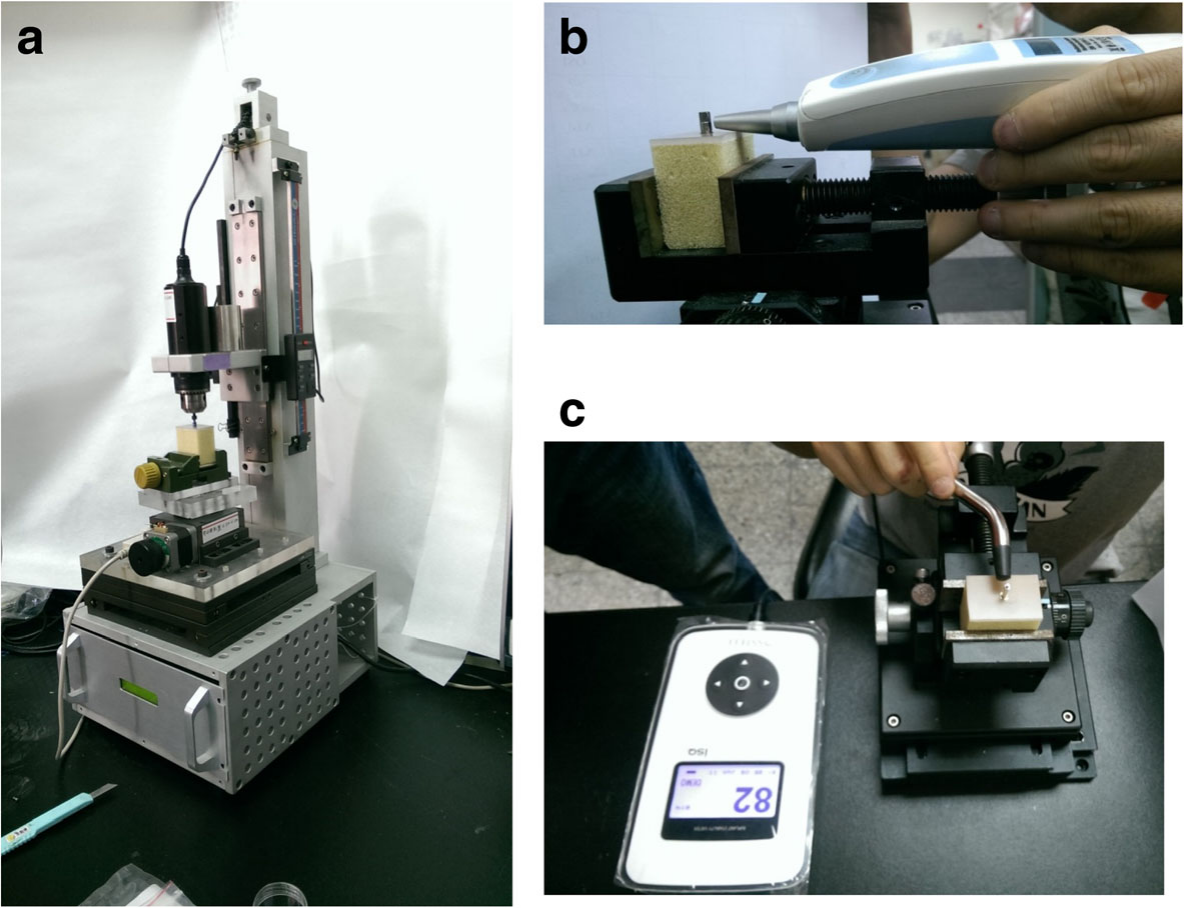
Three indexes of the initial implant stability: (**a**) ITV, (**b**) PTV, and (**c**) ISQ

Pilot holes were drilled into each artificial bone specimen in accordance with the manufacturer’s instructions. The peak ITV was measured for each specimen using a customized torque-rotation machine consisting of a digital torque meter (TQ-8800, Lutron Electronic Enterprise, Taipei, Taiwan), electronic rotation motor, and X-Y table (Fig. 2a). The force when inserting a dental implant into the artificial bone specimen was 14 N. The setting up and procedure used with the experimental machine mainly followed the standard of ASTM F543.

Furthermore, PTV was measured by connecting a temporary abutment (implant temporary hexed cylinder, 3i Implant Innovation) and then measuring the mobility of the implant using the Periotest device (Medizintechnik Gulden, Bensheim, Germany) (Fig. 2b). The tip of the Periotest device was placed perpendicular to the abutment at a distance of 2 mm, and it impacted the implant four times per second for 4 s. The Periotest™ device measures the contact time between the implant and the tapping tip as the single and that single were then transformed to a special value called PTV [23]. In general, a shorter contact time presents a lower value of PTV which indicates that the implant in bone is more stable. The attached microcomputer converted the duration obtained from the measurement cycle to PTV on a scale from −8 (very stable) to +50 (extremely unstable) [23].

Finally, the wireless resonance frequency analyzer Osstell ISQ™ (Osstell ISQ, Osstell AB, Gothenborg, Sweden) was used to measure the ISQ value (Fig. 2c). The smart peg for external hex connection of implant (Type 1, Osstell AB) was placed on the top of the implants. The peg has a magnetic material attached to its upper part. When the probe of the Osstell ISQ instrument is near to the smartpeg, the peg is vibrated by magnetic pulses and then Osstell ISQ instrument can detect the resonance frequency and converted it into a unique value called ISQ. The resonance frequency of the abutment–implant system was assigned a value between 0 and 100 to represent ISQ, where a larger ISQ indicates a higher stability.

### Measuring 3D BIC parameters

The measurement approach of 3D BIC parameters were the same with our previous study. [24] As stated by Stoppie et al.’s study, [25] a thin layer of metal artefact around an metallic implant was existing between the inserted implant and bone in the micro-CT images. [25] In order to prevent the problem of artefact, in this study, the artificial bone specimens and dental implants were scanned separately by micro-CT (SkyScan 1076, Skyscan, Aartselaar, Belgium) at the same resolution (17.2 × 17.2 × 17.2 μm3) but with different scanning voltages and currents (89 kV and 112 μA for dental implants, 49 kV and 149 μA for artificial bones). The obtained micro-CT images of the artificial bones and dental implants were imported into Mimics 15.0 (Materialise, Leuven, Belgium) to create the 3D models of artificial bone and dental implants individually.

**Fig. 3:**
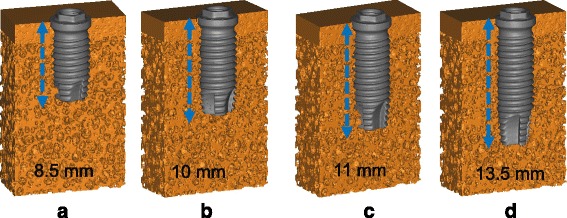
3D models of artificial bone specimens with inserted implants. The lengths of the implants were (**a**) 8.5mm, (**b**) 10mm, (**c**) 11mm, and (**d**) 13.5mm

For the measurement approach of BICA which is the actual area of contact between the bone and implant, the 3D model of dental implant was placed into the 3D model of artificial bone (Fig. 3). Then the intersection area, which is BICA, between the 3D models of implant and artificial bone can be measured by using “Boolean operation” in Mimics.

For the measurement approach of pBICA which is the total exterior surface area of the dental implant within the artificial bone, that can also be calculated in Mimics. Finally, 3D BIC% can be calculated as BICA divided by pBICA. The three BIC parameters were measured four times in different positions for each dental implant.

### Statistical analysis

The values of the three indexes of the primary initial stability (i.e., ITV, PTV, and ISQ) and the three types of 3D BIC parameter (i.e., pBICA, BICA, and 3D BIC%) were summarized as median and interquartile range (IQR) values. The Kruskal-Wallis test was used to compare how the three indexes of primary initial stability and the 3D BIC parameters differed among the implants with four diameters. In addition, post-hoc pairwise comparisons were conducted using the Wilcoxon rank-sum test with Bonferroni adjustment, with the cutoff for statistical significance set at 0.00833 (0.05/6). Spearman correlation coefficients (*R* ^2^; also called coefficients of determination) were used to evaluate significant correlations between the primary implant stability indexes and the 3D BIC parameters. All statistical analyses were performed using SPSS (version 19, IBM Corporation, Armonk, NY, USA).

## Results

### Implant length versus initial implant stability

**Fig. 4:**
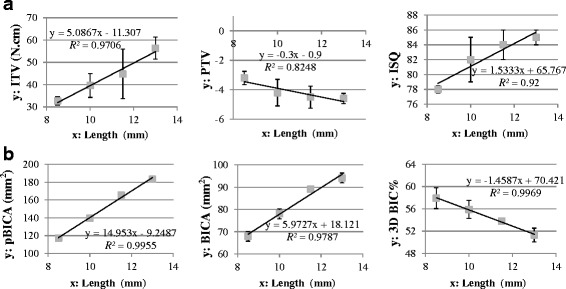
Linear equations and *R* ^2^ values for (**a**) ITV, PTV, and ISQ, and (**b**) pBICA, BICA, and 3D BIC% for implants with four lengths

The median, IQR, maximum, and minimum values of the ITV, PTV, and ISQ initial implant stability indexes are listed in Table 1. All three indexes were significantly influenced (*P* < 0.05) by the implant length. However, post-hoc pairwise comparisons revealed that the implant length affected ITV more than PTV and ISQ (Table 1). The *R* ^2^ values for the correlations between the implant length and the initial implant stability indexes were 0.82–0.97. The correlation was stronger (*R* ^2^ = 0.97) for ITV than for PTV and ISQ (Fig. 4a).

**Table 1:** The values and statistical analyses of three types of initial implant stabilities of various lengths of implants

Length (mm)	ITV (N.cm)				PTV				ISQ			
	Median*	IQR	Max	Min	Median*	IQR	Max	Min	Median*	IQR	Max	Min
8.5	32.70^a^	1.8	34.8	31.2	−3.2 ^a^	0.45	−2.9	−3.85	78.00 ^a^	0.5	80	75
10	39.60^b^	5.3	43	35.2	−4.2 ^ab^	0.9	−3.4	−4.8	82.00^ab^	3	83	80
11.5	44.80^bc^	11.1	52.9	39.3	−4.5^ac^	0.75	−3.8	−5.05	84.00^b^	2	85	83
13	56.40^c^	5	66.7	50.7	−4.6^bc^	0.35	−4.35	−5	85.00^b^	1	85.5	83
*P*†	<0.001				0.019				0.002			

†Kruskal-Wallis test

* Post hoc pairwise comparisons were conducted by exact Mann-Whitney U-Test with the Bonferroni adjustment; medians with the same letter ^a,b,c,d^ are not significantly different at the 0.00833 (0.05/6) level

### Implant length versus 3D BIC parameters

Table 2 lists the median, IQR, maximum, and minimum values of the 3D BIC parameters, and indicates that pBICA, BICA, and 3D BIC% differed significantly (*P* < 0.05) with the implant length. According to the findings of post-hoc pairwise comparisons, the implant length affected pBICA and BICA more than 3D BIC% (Table 2). There were linear correlations between 3D BIC parameters and the implant length, all with *R* ^2^ values higher than 0.97 (Fig. 4b) but with differing slopes. A longer implant increased pBICA and BICA but decreased 3D BIC% (Fig. 4b).

**Table 2:** The values and statistical analyses of three kinds of 3D BICs of various lengths of implants

Length (mm)	pBICA (mm^2^)				BICA (mm^2^)				3D BIC%			
	Median*	IQR	Max	Min	Median*	IQR	Max	Min	Median*	IQR	Max	Min
8.5	117.35 ^a^	0.06	117.39	117.31	67.97 ^a^	2.19	70.33	66.57	57.91 ^a^	1.87	59.93	56.76
10	139.62 ^b^	0.05	139.64	139.57	78.08 ^b^	2.25	79.35	76.31	55.91 ^a^	1.62	56.02	54.65
11.5	165.52 ^c^	0.08	165.56	165.47	89.10 ^c^	0.84	89.38	88.21	53.83 ^b^	0.49	54	53.31
13	183.48 ^d^	0.06	183.54	183.46	94.16 ^d^	2.22	96.13	92.62	51.31 ^c^	1.22	52.39	50.46
*P*†	<0.001				<0.001				0.001			

†Kruskal-Wallis test

* Post hoc pairwise comparisons were conducted by exact Mann-Whitney U-Test with the Bonferroni adjustment; medians with the same letter ^a,b,c,d^ are not significantly different at the 0.00833 (0.05/6) level

### 3D BIC parameters versus initial implant stability

**Fig. 5:**
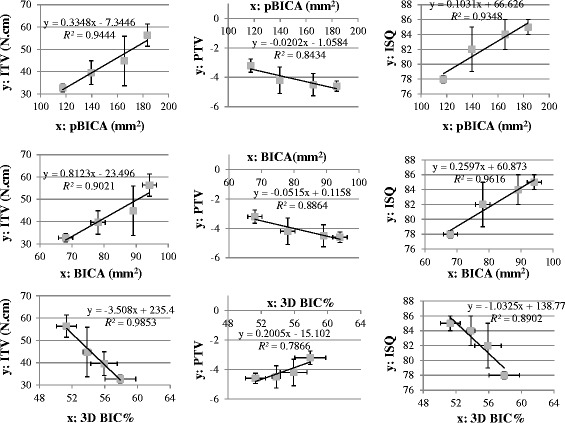
Nine linear equations and *R* ^2^ values for the correlations between primary implant stability indexes (ITV, PTV, and ISQ) and 3D BIC parameters (pBICA, BICA, and 3D BIC%)

Fig. 5 shows the correlations between 3D BIC parameters and indexes of the initial implant stability, all of which had *R* ^2^ values higher than 0.78. In general, the *R* ^2^ values were higher for the correlations of 3D BIC parameters with ITV and ISQ (all >0.89) than for those between 3D BIC parameters and PTV.

## Discussion

It has long been considered that using implants that are as long as possible will maximize the initial implant stability [4], since this will increase the implant surface area and BIC. However, the additional length of the implant will only be in contact with trabecular bone, and so this will limit the real increase in the BIC surface area. The better way to confirm of how much BIC area will be added by embeding longer implant in bone would be to obtain quantitative 3D BIC data. Therefore, the present study combined a micro-CT system with professional medical image-processing software to develop 3D BIC parameters, and evaluated them in relationship to the initial implant stability of implants with various lengths.

The results of this study indicated that ITV is the index of the initial implant stability that seems to be most closely related to changes in implant length. A longer implant is considered to enhance ITV by providing a larger surface area of the implant in contact with trabecular bone, which might provide a greater mechanical resistance against the applied torque. Althoug insertion torque is a destructive measurement that can not be practised in clinic repeatedly, a high ITV indicates that the extent of micromotion between the implant and bone is reduced [26] and that the implant is mechanically more stable [27]. Not only implant length, but bone density [28]and cortical bone thickness [28] has been found by other studies could increase ITV.

The other indexes of the initial implant stability (PTV and ISQ) did not vary clearly with the implant length. These findings are consistent with Degidi et al. [9] reporting a weak correlation between ISQ and implant length, and implant length has lower influence on ISQ as compared to implant diameter. Even though there are different opinions regarding whether the implant length influences ISQ [6], those previous in vitro experiments used solid artificial polyurethane bone block to mimic the trabecular bone. Since it is known that trabecular bone has a porous structure unlike cortical bone, the use of solid specimens to mimic trabecular bone might exaggerate the contact area between implant and bone if longer implant was placed. In the present study, the use of porous trabecular bone specimens showed that there were only significant differences in ISQ values between implants with lengths of 8.5 and 13 mm and of 8.5 and 11.5 mm.

The 3D BIC parameter measurements indicated that pBICA was highest for the longest implant (13 mm long). This is expected since the value of pBICA is directly correlated with the area of the implant surface, and so a longer implant should exhibit a larger surface area of implant. Also, a longer implant also has a larger area of the implant surface in contact with porous trabecular bone, and that would also increase BICA.

However, it was also apparent that using a longer implant reduced 3D BIC%. This difference relative to the findings for BICA and pBICA might be related to 3D BIC% being defined as BICA divided by pBICA. Increasing the implant length only increases the area of the implant surface in contact with trabecular bone, which has a porous structure, and so the values of pBICA and BICA will not increase proportionally, resulting in the change in 3D BIC% differing from those in pBICA and BICA.

Linear regressions between 3D BIC parameters and initial implant stability indexes were evaluated in this study (Fig. 5). The Spearman coefficient was highest (*R* ^2^ > 0.98) for the correlation between 3D BIC% and ITV, which is consistent with Capparé et al. [7] finding that a significant linear correlation was existed between the two-dimensional (2D) BIC% and ITV. It appears that either 3D BIC% or 2D BIC% might be usefully assessed from the variation in ITV. For the linear regression models related to pBICA, the value of *R* ^2^ was high (>0.9) for the correlation between pBICA and ITV or ISQ, which is consistent with the findings of Hong et al. [6]. They found that ITV and ISQ were proportional to the total surface area of the implant fixture, with *R* ^2^ also higher than 0.9. Nevertheless, in our study *R* ^2^ was also high for the linear relationship between BICA and ITV (*R* ^2^ > 0.9) or ISQ (*R* ^2^ > 0.95). It seems that both the total implant surface area and the implant surface area in contact with bone may be good indexes for predicting the initial stability of a dental implant.

The main limitation of this study was the use of artificial foam bone specimens as testing samples, rather than real human bone. The use of artificial bone samples has some advantages, such as consistent porosities of the cancellous bone models as well as the material properties being similar to those of human bone, which avoids the differences between individual human subjects. However, the use of cadavers still needs to be considered in future studies. Another limitation was the smallness of the sample size, comprising only five specimens in each group. Future cadaveric studies might be considered to include more specimens in order to reduce errors caused by individual differences.

## Conclusions

Within the limitations of this study, it is possible to draw the following conclusions. Firstly the implant length had a greater effect on ITV than on ISQ and PTV, and pBICA and BICA increased while 3D BIC% decreased for longer implants. The *R* ^2^ values in linear regression analyses were higher for the correlations between 3D BIC parameters and implant length than for those between indexes of the primary implant stability and the implant length. Finally, the goodness of fit (as reflected by *R* ^2^) was better for linear relationships between 3D BIC parameters and ITV or ISQ. That might reveal that all three 3D BIC parameters (i.e., pBICA, BICA and 3D BIC%) are reflected in variations in ITV and ISQ, which may be helpful in the treatment plans that include the possibility of selecting longer implants.

## Abbreviations

3D BIC%: 3D BIC percentage
BIC: Bone-to-implant contact
BICA: BIC area;
CBCT: Cone-bean computer tomography
IQR: Interquartile range
ISQ: Implant stability quotient
ITV: Insertion torque value
micro-CT: Microcomputed tomography
pBICA: Potential BIC area
PTV: Periotest value
R2: Correlation coefficients
